# Pangenomic analysis of *Wolbachia* provides insight into the evolution of host adaptation and cytoplasmic incompatibility factor genes

**DOI:** 10.3389/fmicb.2023.1084839

**Published:** 2023-02-03

**Authors:** Bo Liu, Ye-Song Ren, Cheng-Yuan Su, Yoshihisa Abe, Dao-Hong Zhu

**Affiliations:** ^1^Laboratory of Insect Behavior and Evolutionary Ecology, College of Life Sciences, Central South University of Forestry and Technology, Changsha, China; ^2^Guangdong Laboratory of Lingnan Modern Agriculture, Genome Analysis Laboratory of the Ministry of Agriculture and Rural Affairs, Agricultural Genomics Institute at Shenzhen, Chinese Academy of Agricultural Sciences, Shenzhen, China; ^3^Faculty of Social and Cultural Studies, Kyushu University, Fukuoka, Japan

**Keywords:** *Wolbachia*, host adaptation, cytoplasmic incompatibility, evolution, genomics

## Abstract

**Introduction:**

The genus *Wolbachia* provides a typical example of intracellular bacteria that infect the germline of arthropods and filarial nematodes worldwide. Their importance as biological regulators of invertebrates, so it is particularly important to study the evolution, divergence and host adaptation of these bacteria at the genome-wide level.

**Methods:**

Here, we used publicly available *Wolbachia* genomes to reconstruct their evolutionary history and explore their adaptation under host selection.

**Results:**

Our findings indicate that segmental and single-gene duplications, such as DNA methylase, bZIP transcription factor, heat shock protein 90, in single monophyletic *Wolbachia* lineages (including supergroups A and B) may be responsible for improving the ability to adapt to a broad host range in arthropod-infecting strains. In contrast to A strains, high genetic diversity and rapidly evolving gene families occur in B strains, which may promote the ability of supergroup B strains to adapt to new hosts and their large-scale spreading. In addition, we hypothesize that there might have been two independent horizontal transfer events of *cif* genes in two sublineages of supergroup A strains. Interestingly, during the independent evolution of supergroup A and B strains, the rapid evolution of *cif* genes in supergroup B strains resulted in the loss of their functional domain, reflected in a possible decrease in the proportion of induced cytoplasmic incompatibility (CI) strains.

**Discussion:**

This present study highlights for reconstructing of evolutionary history, addressing host adaptation-related evolution and exploring the origin and divergence of CI genes in each *Wolbachia* supergroup. Our results thus not only provide a basis for further exploring the evolutionary history of *Wolbachia* adaptation under host selection but also reveal a new research direction for studying the molecular regulation of *Wolbachia*- induced cytoplasmic incompatibility.

## Introduction

*Wolbachia* belongs to the Anaplasmataceae in Rickettsiales, and its members are common intracellular symbionts of arthropods and nematodes (Wolbach and Hertig, 1924). *Wolbachia* species not only have a wide host range, including species of *Culex* (Werren et al., 2008), *Aedes* (Trpis et al., 1981), *Drosophila* (Teixeira et al., 2008; Klasson et al., 2009), parasitic wasps (Monnin et al., 2017) and a variety of lepidopteran pests (Delgado and Cook, 2009; Ju et al., 2020), but also exert various regulatory effects on their hosts. Not surprisingly given this high incidence and wide host range, the *Wolbachia* clade exhibits high genetic diversity (Zug and Hammerstein, 2015; Detcharoen et al., 2019; Kaur et al., 2021).

*Wolbachia* strains are distributed in several large clades referred to as ‘supergroups’ that have likely diverged over hundreds of millions of years (Bordenstein and Rosengaus, 2005; Lo et al., 2007; Glowska et al., 2015; Gerth and Bleidorn, 2016; Lefoulon et al., 2020). However, these large groups could in principle take on species status, which is a matter of ongoing debate (Shamayim et al., 2015; Gerth, 2016; Shamayim et al., 2016; Bleidorn and Gerth, 2018). *Wolbachia* classification is based on molecular data and loci that are regularly employed for strain discrimination at various levels, such as the 16S rRNA gene, five multilocus sequence typing loci (MLST) and the *Wolbachia* surface protein gene (*wsp*). A total of 14 *Wolbachia* supergroups (designated A–O) have been described in different host taxa. Most arthropod-associated *Wolbachia* strains are defined as belonging to supergroups A and B (Baldo et al., 2006; Lo et al., 2007), nematode-infecting strains are defined as belonging to supergroups C and D (Bandi et al., 1998). Supergroups E and F have been found in, arthropods (Czarnetzki and Tebbe, 2004; Panaram and Marshall, 2007) and nematodes (Fenn and Blaxter, 2004). Supergroup G is restricted to spiders (Rowley et al., 2004), supergroup H has been identified in association with dampwood termites (Bordenstein and Rosengaus, 2005), and supergoups M and N have been found in aphids (Augustinos et al., 2011; Wang et al., 2014).

In recent years, with the rapid development of technologies for DNA sequencing and extracting DNA from whole insect hosts, the whole-genome sequencing of *Wolbachia* has been realized (Darby et al., 2012). The wMel strain of *Drosophila melanogaster* was the first *Wolbachia* strain to have its full genome sequence published (Wu et al., 2004). The genome size of the wMel strain is approximately 1.27 Mb and contains a large number of repeated sequences and mobile elements, which is rare among intracellular species. In contrast, the wBm strain hosted by filarial nematodes contains no prophage and fewer repeat sequences (Foster et al., 2005). To date, many *Wolbachia* strain genomes have been released in the National Center for Biotechnology Information (NCBI) database, which provides data support for revealing the evolution of host adaptation and regulation of host interactions between strains and their hosts.

The predominant mode of *Wolbachia* transmission within a species occurs *via* the egg cytoplasm, resulting in vertical transmission. Due to this transmission pattern, *Wolbachia* exerts regulatory effects on host reproduction, the most common of which changing the sex ratio of the host population (Engelstaedter and Hurst, 2009). *Wolbachia* was first discovered in the reproductive tissues of *Culex pipiens* (Werren et al., 2008), in which the bacterium showed cytoplasmic incompatibility with its host (Yen and Barr, 1971). *Wolbachia* has since been found to have other reproductive regulatory functions, such as male killing (Stouthamer et al., 1999), feminization (Rousset et al., 1992) and parthenogenesis (Stouthamer et al., 1990), making it a hot topic of research. *Wolbachia* manipulates insect reproduction by enhancing its inheritance through the female germline. The most common mode of reproductive manipulation is the induction of cytoplasmic incompatibility (CI) (Yen and Barr, 1971; Hunter et al., 2003), in which eggs from uninfected females fail to develop when fertilized by sperm from *Wolbachia*-infected males, which results in embryonic lethality in crosses between infected males and uninfected females. Based on comparative and transgenic approaches, previous studies have shown that two differentially transcribed, codiverging genes in the eukaryotic association module of prophage WO from *Wolbachia* strain wMel recapitulate and enhance cytoplasmic incompatibility (Lindsey et al., 2018). Another study revealed that CI-like embryonic lethality could be recapitulated in *Drosophila melanogaster* males through the transgenic coexpression of homologous transgenes *cifA* and *cifB*, encoded by the wPip strain of *Wolbachia*, which naturally infects *Culex mosquitoes* (Beckmann et al., 2017). In previous research, the CI factors *cifA* (locus WD0631) always encoded directly upstream of *cifB* (locus WD0632) in the genome of wMel strain (LePage et al., 2017). *In vitro* functional analyses revealed that *cifB* encodes deubiquitylase activity, and *cifA* encodes a protein that binds *cifB* (Beckmann et al., 2017). Mutating the predicted catalytic residue in the deubiquitylating domain of *cidB* results in a loss of the CI-like function in transgenic flies (Beckmann et al., 2017). The presence of the two genes within prophage WO has implications for the transmission of these genes since temperate phage WO exhibits frequent lateral transfers between *Wolbachia* (Bordenstein and Wernegreen, 2004; Chafee et al., 2010). Whether the origin and evolution of these genes are important for their function remains an open question.

In the present research, we aim to reconstruct the evolutionary history, investigate the host adaptation-related evolution and explore the origin and divergence of CI genes in each *Wolbachia* supergroup based on the analysis of gene family expansion, genetic diversity and syntenic relationships in comparisons of 57 *Wolbachia* genomes. Our study not only provides guidance regarding the coevolution of intracellular symbionts and hosts but also generates new ideas about the origin and evolution of key genes involved in cytoplasmic incompatibility.

## Materials and methods

### Obtaining genome sequences in *Wolbachia* strains

The genome sequences used in this study were downloaded from the National Center for Biotechnology Information (NCBI)^1^ up to May 2020. We filtered the genomes according to the following criteria: (1) we filtered out *Wolbachia* strains without available host information; (2) when the strains had the same name, we retained the more recently submitted genome version; and (3) the genome sequences of strains without predictive genes were filtered out. Finally, a total of 57 *Wolbachia* strain genomes were analyzed in this study. The NCBI accession numbers of all *Wolbachia* genome sequences are given in Supplementary Table S1.

### Gene function annotation and enrichment analysis

Gene functional annotation was performed by aligning the corresponding protein sequences to the NCBI nonredundant (NR), Universal Protein (UniProt) (Wu et al., 2006), Evolutionary Genealogy of Genes: Nonsupervised Orthologous Groups (eggNOG) (Huerta-Cepas et al., 2017) and Kyoto Encyclopedia of Genes and Genomes (KEGG) databases (Kanehisa et al., 2004) by using BLASTP v2.3.0+ with an E-value cut-off of 10^−5^. InterProScan v2.0 (Quevillon et al., 2005) was used to assign preliminary Gene Ontology (GO) terms, Pfam domains and IPR domains to each gene. The enrichment analysis of GO and KEGG pathways was performed using the online OmicShare platform^2^.

### Phylogenetic analysis

Orthologous and paralogous gene families of 57 *Wolbachia* strains, one *Ehrlichia canis* str. YZ-1 (PRJNA429059) and one *Anaplasma marginale* str. Florida (PRJNA16369) were assigned by OrthoFinder v0.4 (Emms and Kelly, 2015) with the parameters “-f –t 30.” The orthologous groups that contained only one gene in each strain were selected to construct the phylogenetic tree. The protein sequences of each orthologous group were independently aligned with MUSCLE v3.8.31 (Edgar, 2004) with the parameters “-maxiters 16” and then concatenated into one supersequence. The phylogenetic tree was constructed based on maximum likelihood (ML) using PhyML v3.0 (Guindon et al., 2010) with the best-fit model (HIVb+I + G + F) that was estimated by ProtTest3 (Darriba et al., 2011). Node support was estimated with 1,000 bootstrap replicates.

The nucleotide sequences of five housekeeping genes (*gatB*, *coxA*, *hcpA*, *ftsZ*, and *fbpA*) that were downloaded from the PubMLST database^3^ were aligned to the gene sets of all strains by using BLASTP v2.3.0 with the parameter setting of 10^−20^. Then, the maximum likelihood tree was constructed by using PhyML v3.0 with the parameters “-d aa –m LG –c 4.” Node support was estimated with 1,000 bootstrap replicates.

To infer the divergence times of different *Wolbachia* supergroups in the phylogeny, divergence time estimates were calculated using r8s v1.8.1 (Sanderson, 2003) with the parameters “-b -f” by fitting branch lengths of an ML tree using penalized likelihood and a smoothing parameter of 8, chosen as optimal by cross-validation. The two secondary calibration points obtained from Michael et al. (2016), where ~217 million years ago (Mya) was the split time of wAu and wNo.

### Gene family expansion and contraction analysis

To determine the change in orthologous group members of *Wolbachia* strains during evolution, the analysis of gene gains and losses was conducted using CAFÉ v3.0 (De Bie et al., 2006), in which orthologous group change was simulated using a stochastic birth and death model. The optimal lambda parameter was automatically determined independently. The orthologous groups with *p* values <0.05 were defined as rapidly evolving families in the CAFÉ results. We also used the *z*-test (*p* < 0.05) to identify the expanded orthologous group in each *Wolbachia* supergroup based on the gene numbers. We used OmicShare and WEGO v2.0 (Ye et al., 2018) to analyse the functional enrichment of expansive orthologous groups.

### Genome evolution analysis

Genomic synteny fragments were identified with MCscanX (2012) (Wang et al., 2012), requiring at least five gene pairs per collinear block. Then, we used the duplicate_gene_classififier (2012) of MCscanX to identify duplicated genes and classified the origins of the genes into different types, including segmental, tandem, proximal and dispersed duplications. We employed the inter- and intrasyntentic gene pairs to calculate synonymous mutation (*K_s_*) values by using KaKs_Calculator v2.0 (Wang et al., 2010) with the parameter “-c 1 -m MS.” The orthologous groups that contained only one gene for each strain were selected. Then, the nucleotide diversity of the single-copy genes within each *Wolbachia* supergroup was calculated by using DnaSP v6.12.03 (Rozas et al., 2017). The identity of genome-wide nucleotide sequences in each pair of strains was determined by using Mummer v3.23 package (Kurtz et al., 2004; delta-filter -i 75 -l 1,000 and show-coords –r –c -l).

### Analysis of *cif* genes in each *Wolbachia* strain

The *cifA* (GeneID: 69724995 and GeneID: 61803217) and *cifB* (GeneID: 69724996 and GeneID: 61803216) gene sequences used in this study were downloaded from the National Center for Biotechnology Information (NCBI)^4^. To identify the *cifA and cifB* genes in each *Wolbachia* strain, we used the two *cif* gene sequences to align to the gene set of each strain by using BLASTN v2.3.0+ with the parameters word_size = 4 and Evalue = 10. Then, to avoid missing the *cif* genes of each strain, synteny analysis was performed between the prophage WO genome and each *Wolbachia* genome to find more *cif* genes. To identify the divergence times of the *cif* genes in *Wolbachia* strains, we used the *cifA* and *cifB* gene pairs within supergroups A and B, respectively, to calculate the synonymous (*K_s_*) and nonsynonymous (*K_a_*) mutation rates by using KaKs_Calculator v2.0 with the parameters “-c 1 -m MS.” The nucleotide diversity (*π*) and genetic distance of *cif* genes within supergroups A and B were calculated by using DnaSP v6.12.03. The motifs of the *cifA* and *cifB* gene sequences in each *Wolbachia* strain were analysed by using MEME^5^ with 10 motifs should MEME find. We used JCVI (Tang et al., 2015) to construct the local syntenic relationships of each gene in the supergroup A strain.

## Results

### The larger genome sizes of supergroups A and B are mostly derived from small-scale gene duplications

**Figure 1:**
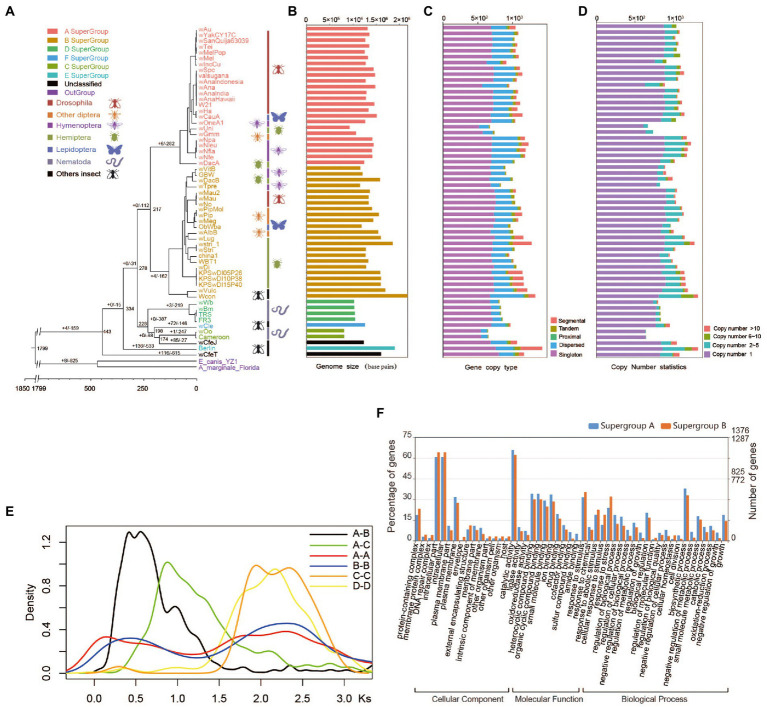
Evolution of *Wolbachia* genomes. **(A)** Paleohistory of *Wolbachia* with *Ehrlichia canis* and *Anaplasma marginale* as outgroups. The number at the nodes represents the divergence time of species. **(B)** Genome sizes of *Wolbachia* strains. **(C)** The different gene copy types in *Wolbachia* strains were identified and classified by using the software duplicate_gene_classififier. The gene duplication types were classified as segmental, tandem, proximal and dispersed duplications. Segmental indicates anchor/collinear genes in syntenic blocks; tandem indicates continuous repeat; proximal indicates in nearby chromosomal region but not adjacent; dispersed indicates other modes than segmental, tandem and proximal. **(D)** Numbers of gene copies. **(E)** *Ks* distribution of syntenic orthologues from four *Wolbachia* supergroups. The y-axis shows the ratio of gene pairs in the syntenic block. **(F)** Functional enrichment of all duplicated genes in supergroups A and B according to Gene Ontology (GO) classification.

To assess the paleohistory of *Wolbachia* strains, we downloaded 57 available genomic sequences of *Wolbachia*, including sequences of supergroups A, B, C, D, E and F as well as the unclassified *Wolbachia* supergroup, and performed a comparative genomic investigation with *Ehrlichia canis* and *Anaplasma marginale* as outgroups (Supplementary Table S1). Among these genomes, a total of 60,932 genes were clustered into 2,381 orthologous groups containing 109 single-copy orthologues. The phylogenetic trees showed that 24 and 23 *Wolbachia* strains were clustered into supergroups A and B, respectively. Supergroups A and B shared a common ancestor (Figure 1A) corresponding to the topological structure based on housekeeping genes (Supplementary Figure S1A). The estimated divergence time analysis indicated that *Wolbachia* diverged from the two outgroup genera (*Anaplasma* and *Ehrlichia*) ~1799 million years ago (Mya). Arthropod-infecting *Wolbachia* supergroups A and B strains were reciprocally monophyletic and diverged from their common ancestor 217 Mya. supergroups C, D, F, and wCfeJ formed a monophyletic group, which corresponded to previously published results (Gerth et al., 2014; Michael et al., 2016). Arthropod-infecting *Wolbachia* strains diverged from nematode-infecting *Wolbachia* strains 278 Mya (Figure 1A).

The nature and relative importance of the molecular mechanisms and evolutionary forces underlying genome size variation have been the subject of intense research and debate (Petrov, 2001; Elliott and Gregory, 2015). A number of correlative associations between genome size and phenotypic traits suggest that natural selection and adaptive processes also shape genome size evolution (Cavalier-Smith, 1982; Andrews et al., 2009; Wright et al., 2014; Ellegren and Galtier, 2016). The present study showed distinct differences in genome size between *Wolbachia* supergroups, indicating that the average genome size of arthropod-infecting *Wolbachia* strains was 1.47 times larger than that of nematode-infecting *Wolbachia* strains (*t* test, *p* = 5.82E^−06^; Figure 1B). Previous studies have documented that the differential expansion, accumulation and removal of transposable element (TE) sequences are major determinants of genome size variation between *Wolbachia* strains wBm and wMel (Foster et al., 2005). However, we found that the larger genome size of arthropod-infecting *Wolbachia* strains, in which the gene numbers were significantly greater than those in nematode-infecting *Wolbachia* strains, was due not only to an increase in repeat sequences but also to gene duplications (Figures 1B–D; Supplementary Figure S1B). Based on gene copy number analysis, 684, 717, 692 and 547 genes (on average) were assigned to single-copy genes in supergroups A, B, C and D, respectively. Unexpectedly, it was found that approximately 33.9% of the total genes in arthropod-infecting *Wolbachia* strains were likely produced through small-scale gene duplication events (Figure 1C; Supplementary Figure S1C), dominated by genes showing 2–5 copies (Figure 1D; Supplementary Figure S1D), which was significantly greater than the number in supergroups C (16.9%) and D (15.6%). Among these duplicated genes in *Wolbachia* supergroup A, an average of 280 dispersed duplicated genes were found, which was similar to the number in supergroup B (242 genes) but significantly higher than that in supergroups C and D (85 and 118 genes, respectively).

To study the history of gene duplications, we identified the genes showing inter- and intraspecies homology between each supergroup and then calculated the synonymous mutation rates (*K_s_*) of the syntenic fragments of orthologous pairs. Apparent *K_s_* peaks were observed in all of the four supergroups (A, B, C and D) (Figure 1E), which have a complex history of duplication involving two small-scale gene duplications instead of a whole-genome duplication. According to a *K_s_* value of less than 1, 34.1 and 59.1% segmentally duplicated gene pairs were identified within supergroups A and B, respectively, indicating that a recent duplication event occurred during the divergence of supergroup A and B strains. Single-gene duplication events with a peak of *K_s_* = 1.75–3, dominated by dispersed gene duplications, were shared by the common ancestor of each supergroup. In contrast to the evolutionary history of supergroups A and B, no evidence of a recent gene duplication event was detected in supergroups C and D based on the *K_s_* distribution (Figure 1E).

All of duplicated genes identified in supergroups A and B were significantly enriched in the following functional categories based on the GO and KEGG analyses: catalytic and hydrolase activity, nitrogen compound metabolism, transcription factor, response to stimulus and chemical (Figure 1F; Supplementary Figure S2), such as DNA methylase, bZIP transcription factor, heat shock protein 90, DNA mismatch repair protein MLH1, cysteine protease and so on. In addition, the enrichment of ABC transporters in supergroups A and B was mainly due to the duplication of ATP-binding cassette subfamily A/B/D/G genes (EC:7.6.2.2 and EC:7.6.2.4). The gene encoding cucurbitadienol synthase (EC:5.4.99.33), 5-phosphonooxy-L-lysine phospho-lyase (EC:4.2.3.134), vanillin aminotransferase (EC:2.6.1.119) and vanillin aminotransferase (EC:2.6.1.119) were also expansion in the supergroups A and B, in which the cucurbitadienol synthase was not present in the supergroups C and D. This demonstrated that extensive gene fractionation occurred during the evolutionary history of arthropod-infecting *Wolbachia* strain genomes, which promoted the retention of essential genes for survival and host adaptation.

### Gene duplication enhances the host adaptation of arthropod-infecting *Wolbachia* strains

**Figure 2:**
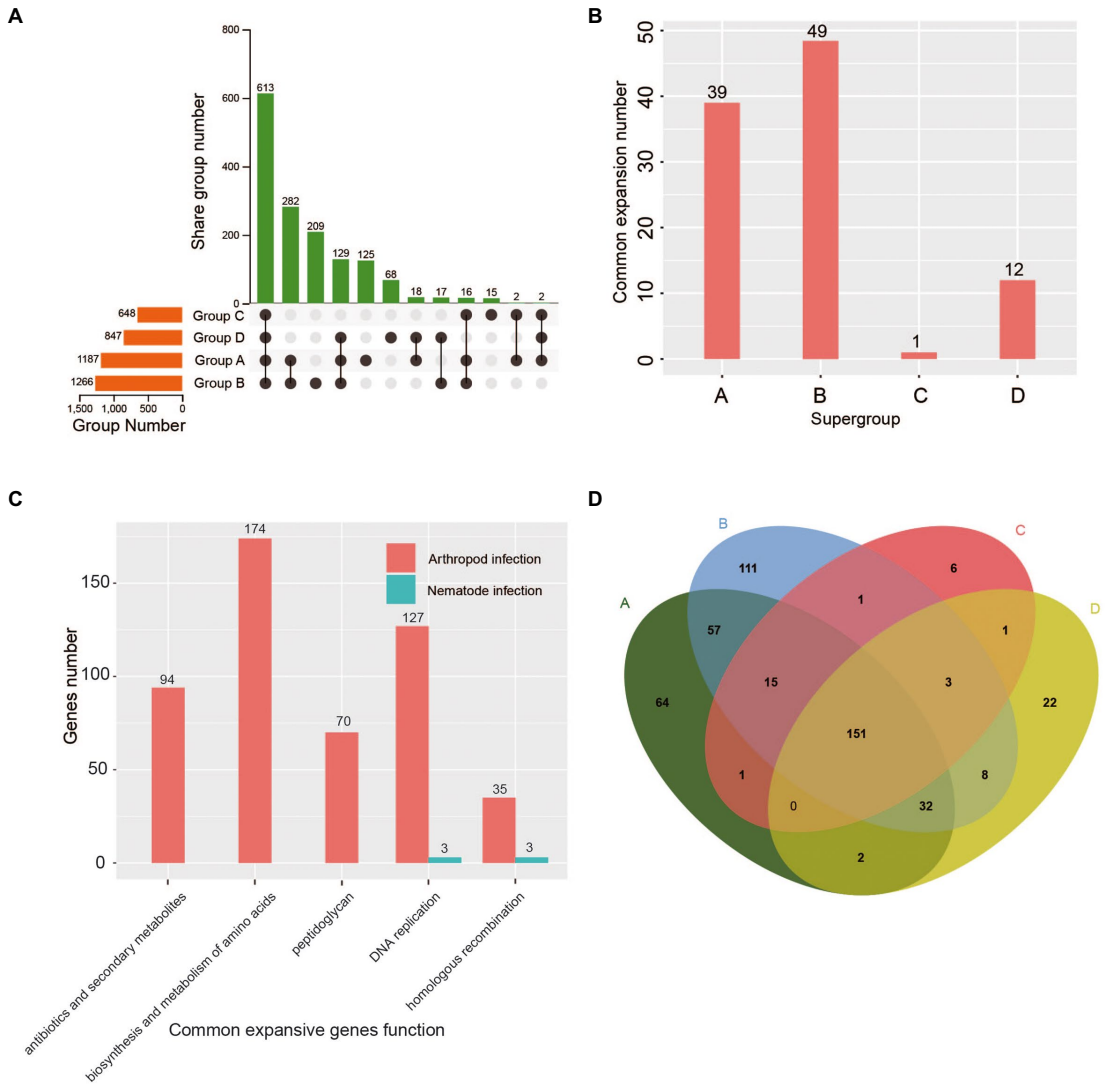
Comparison of gene families and functional enzyme expansions among the four *Wolbachia* supergroups (A, B, C, and D). **(A)** Unique and shared orthologous groups between and among the four supergroup genomes. **(B)** Number of coexpansive orthologous groups within each supergroup. **(C)** KEGG functional enrichment analysis of coexpanded genes in arthropod-infecting and nematode-infecting *Wolbachia* strains. **(D)** Venn diagram showing the number of unique and shared functional enzymes between and among the four supergroups, A, B, C and D.

In this study, 2,381 orthologous groups were found in 57 *Wolbachia* strains, in which the average number of orthologous groups in arthropod-infecting *Wolbachia* supergroups (A and B) was significantly higher (1.6 times) than that in nematode-infecting *Wolbachia* supergroups (C and D) (Figure 2A). Otherwise, the supergroup-specific orthologous groups displayed a similar variation pattern, in which the average number of orthologous groups in arthropod-infecting *Wolbachia* was 4.6 times higher than that in nematode-infecting groups (Figure 2A).

Unexpectedly, the average number of expanded orthologous groups was significantly greater in arthropod-infecting *Wolbachia* groups than in nematode-infecting groups (Supplementary Figure S3). Among those expanded orthologous groups, the number of orthologous groups that underwent common expansion within arthropod-infecting *Wolbachia* strains was significantly higher than that in nematode-infecting *Wolbachia* strains (A/C *p <* 0.001, A/D *p <* 0.001, B/C *p <* 0.001, B/D *p <* 0.001; Figure 2B). In contrast to nematode-infecting *Wolbachia*, the function of common expansive genes in arthropod-infecting strains was primarily enriched in DNA replication, homologous recombination and the biosynthesis and metabolism of amino acids, peptidoglycan, antibiotics and secondary metabolites (Figure 2C).

The same conclusion was reached for functional enzymes, where an average of 350 kinds of enzymes were identified in the arthropod-infecting supergroup, which was significantly higher than the number in the nematode-infecting supergroup (Figure 2D). The number of supergroup-specific enzymes in arthropod-infecting *Wolbachia* strains was also significantly greater than that in nematode-infecting strains (A/C *p <* 0.001, A/D *p <* 0.001, B/C *p <* 0.001, B/D *p <* 0.001; Figure 2D).

The above evidence indicated a large amount of gene over retention, which was related to the synthesis and metabolism of amino acids and other important compounds and has previously been observed in the genomes of arthropod-infecting *Wolbachia* strains, improving the ability to adapt to a broad host range (Gerth et al., 2014). In contrast to arthropod-infecting *Wolbachia*, the more host-specific supergroups C and D have established long-lasting mutualistic relationships with their hosts, leading to a stable state of the genome that does not require large amounts of gene duplication.

### Adaptive evolution of supergroup B strains to a broad host range

**Figure 3:**
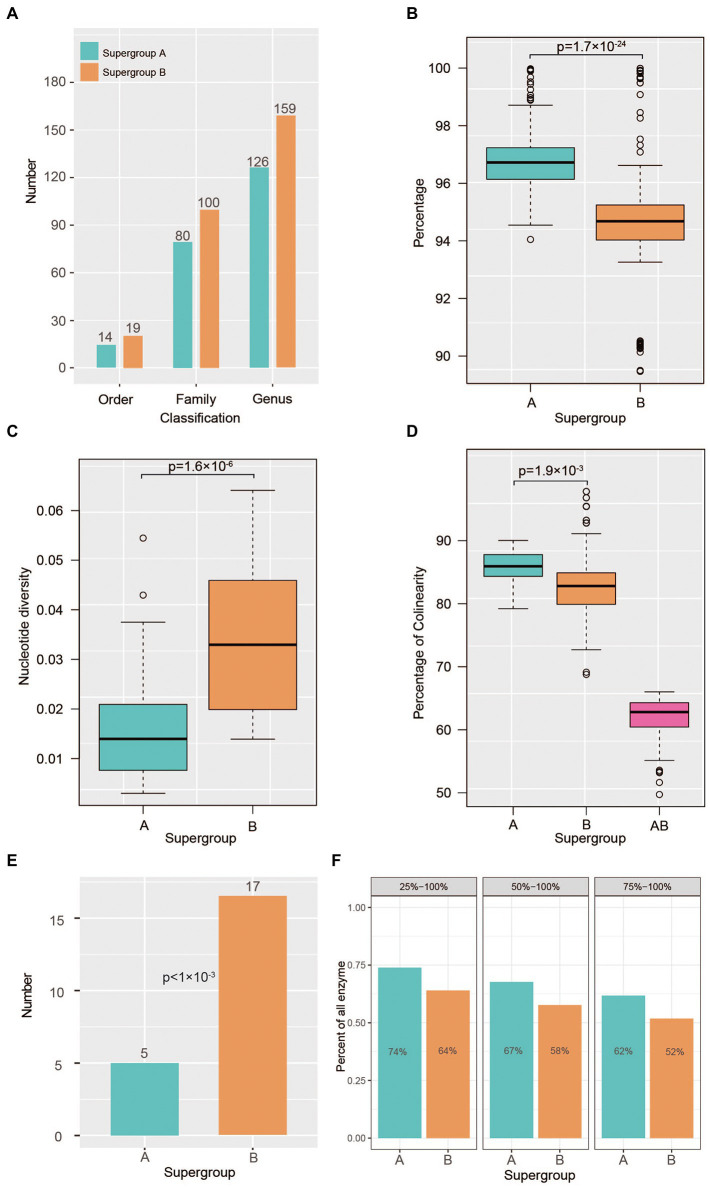
Rapid genomic evolution in *Wolbachia* supergroup B strains. **(A)** Statistics of host species numbers among supergroups A (green box) and B (orange box) based on studies in the last 20 years. **(B)** Analysis of whole-genome identity within supergroups A and B. **(C)** Nucleotide diversity (Pi) within supergroups A (green box) and B (orange box). **(D)** Percentage of syntenic genes of each pair of strains within supergroups A (green box) and B (orange box). And percentage of syntenic genes of each pair of strains between supergroups A and B (purple box) **(E)** Number of rapidly evolving gene families in the ancestors of supergroups A (green box) and B (orange box). Gene families with a *p* value <0.05 were defined as rapidly evolving families in the CAFÉ results. **(F)** Percentage of shared functional enzymes within supergroups A (green box) and B (orange box), where 25–100% indicates that more than 25% of *Wolbachia* strains in each supergroup shared the same functional enzyme; 50–100% indicates that more than 50% of *Wolbachia* strains in each supergroup shared the same functional enzyme; and 75–100% indicates that more than 75% of *Wolbachia* strains in each supergroup shared the same functional enzyme.

According to previous studies on *Wolbachia* strains conducted in the last 20 years, supergroup A strains have infected approximately 162 arthropods, including members of 14 orders, 80 families and 126 genera (Figure 3A; Supplementary Table S2). In stark contrast, the supergroup B strains present a wider host range, infecting 185 arthropods of 19 orders, 100 families and 185 genera (Figure 3A; Supplementary Table S2). To investigate the adaptive evolution of the host range of *Wolbachia* strains, the genetic diversity within supergroups A and B was assessed in this study. In the whole-genome alignments used to analyse the ingroup sequence identity estimated from all *Wolbachia* strains, high conservation was detected in supergroup A, in which the sequence identity between the two strains was 97% on average, ranging from 94 to 99%. In contrast, sequence identity at the genome level between the two supergroup B strains presented significant variance compared with that in supergroup A (*t* test, *p* = 1.7E^−24^; Figure 3B). The further analysis of nucleotide diversity (*π*) among single-copy genes within supergroups A and B revealed a similar variation pattern, in which the π value of conserved genes within supergroup A strains was significantly lower than that within supergroup B strains (*t* test, *p* = 1.6E^−6^; Figure 3C). The results showed that the π values of genes between the supergroup A strains varied from 0.00187 to 0.04841 (0.01639 on average), whereas it varied from 0.01391 to 0.06408 (0.0342 on average) in the B strains.

To investigate the evolutionary dynamics of genome structure, we performed a comparative genomic analysis among supergroup A and B strains using proteins as markers to identify syntenic genes. Within the supergroup A genomes, 85.9% of genes (median value; range 79.2 to 90.0%) showed syntenic relationships between any two strains, whereas 82.8% of genes (range 68.8 to 97.8%) showed syntenic relationships in B strains (*t* test, *p* = 0.0019; Figure 3D). By examining the syntenic relationships between the supergroup A and B strains, we found that the collinearity ratio between the two supergroups was low (Figure 3D), although it was significantly higher than that of nematode-infecting *Wolbachia* strains (Supplementary Figure S4), which was reasonable considering their phylogenetic distance.

According to the CAFÉ analysis, we found that the number of rapidly evolving gene families was significantly higher in the ancestors of supergroup A than in those of supergroup B (*p* < 0.001; Figure 3E). Based on the gene functional analysis, these rapidly evolving gene families were associated with multiple metabolism-related pathways (Supplementary Figure S5), such as acarbose and validamycin biosynthesis, biosynthesis of vancomycin group antibiotics, polyketide sugar unit biosynthesis. Furthermore, we analysed the number of enzymes shared among *Wolbachia* strains, and it was found that there were significantly more kinds of common enzymes in supergroup A strains than in supergroup B strains (Figure 3F), indicating that the rapid evolution of supergroup B strains has enabled them to retain more enzymes for adaptation to a broad host range.

### Origin and evolution of *Wolbachia* cif genes

**Figure 4:**
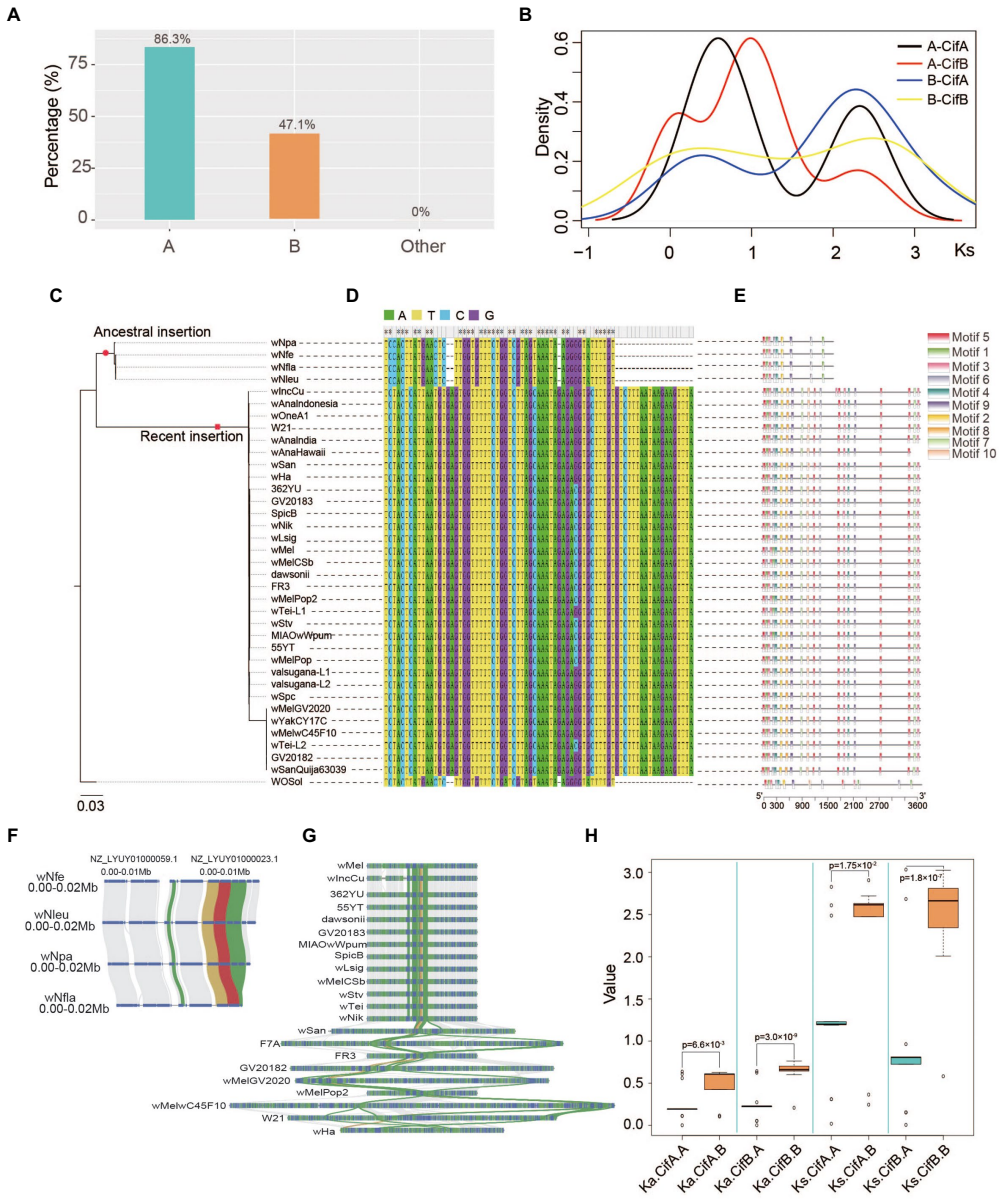
Evolution and diversity of *Wolbachia cif* genes in supergroups A and B. **(A)** Proportion of *Wolbachia* strains containing two *cif* genes in different supergoups. Supergroup A (green box), Supergroup B (orange box) and the other supergroups (red box) **(B)** *Ks* distribution of *cif* genes in *Wolbachia* supergroups A and B. A-CifA and A-CifB indicate the *Ks* distribution of *cifA* and *cifB* genes, respectively, between two strains in supergroup A. B-CifA and B-CifB indicate the *Ks* distribution of *cifA* and *cifB* genes, respectively, between two strains in supergroup B. **(C)** Maximum likelihood tree of *cifB* genes in supergroup A strains that contained both *cif* genes. The red circle represents the ancient *cif* gene insertion event. The red square represents the recent *cif* gene insertion event. **(D)** Multiple sequence alignment of intergenic sequences between two *cif* genes. **(E)** Motif composition of the *cifB* gene-encoding sequence. Each colour box of rectangle represents a different motif. The dials at the bottom indicate the length of the coding sequence. **(F)** Microsyntenic graph of ancient insertions with the same haplotype in *Wolbachia* genomes. The red and yellow lines represent the syntenic relationships of the *cifA* and *cifB* genes, respectively. The green and grey lines represent the syntenic relationships of phage WO and *Wolbachia* genes, respectively. The blue boxes indicate genes in each strain genome. **(G)** Microsyntenic graph of recent insertions with the same haplotype in *Wolbachia* genomes. The red and yellow lines represent the syntenic relationships of the *cifA* and *cifB* genes, respectively. The green and grey lines represent the syntenic relationships of phage WO and *Wolbachia* genes, respectively. The blue boxes indicate genes in each strain genome. **(H)** Comparison of the synonymous mutation rate (*K_s_*) and nonsynonymous mutation rate (*K_a_*) in *Wolbachia* supergroups A and B. ka.cifA. A indicates the *K_a_* values of *cifA* gene between two strains in supergroup A. ka.cifB. B indicates the *K_a_* values of *cifB* gene between two strains in supergroup B, ks.cifA. B indicates the *K_s_* values of *cifA* gene between two strains in supergroup B, ks.cifB. A indicates the *K_s_* values of *cifB* gene between two strains in supergroup A.

To investigate the evolution of *cif* genes in each *Wolbachia* supergroup, a comparative genomic strategy was applied in this study. A literature review focusing on parasitic reproductive modulation by *Wolbachia* showed that a total of 28 CI-inducing *Wolbachia* strains have been identified (Table 1). Among these CI-inducing *Wolbachia* strains, 42.9% (12 of 28) belonged to supergroup A, which was nearly twice the percentage in supergroup B. Notably, based on the retention and deletion analysis of *cif* genes in the genomes of *Wolbachia* strains, approximately 86.3% (44 of 51) of strains contained both *cif* genes in supergroup A, which was significantly higher than the percentage in supergroup B (chi-squared test, *p* < 0.0001; Figure 4A), while none of the *cif* genes were detected in the other supergroups.

**Table 1:** Information of CI-inducing *Wolbachia* strains.

Supergroup	Strain	Host	Reference
A	wMel	*Drosophila melanogaster*	Merot and Charlat (2004), Detcharoen et al. (2021) and Liang et al. (2020)
	wRi	*Drosophila simulans*	Merot and Charlat (2004)
	wHa	*Drosophila simulans*	Merot and Charlat (2004)
	wCobs-BR	*Cardiocondyla obscurior*	Un et al. (2021)
	wCobs-JP	*Cardiocondyla obscurior*	Un et al. (2021)
	Unnamed	*Anopheles moucheti*	Walker et al. (2021)
		*Anopheles demeilloni*	
	wCer2	*Rhagoletis cerasi*	Wolfe et al. (2021)
	wCin2	*Rhagoletis cingulata*	Wolfe et al. (2021)
	wCer2-L2	*Ceratitis capitata*	Morrow et al. (2020)
	wLrr	*Haematobia irritans irritans*	Madhav et al. (2020)
	Unnamed	*Habrobracon hebetor*	Nasehi et al. (2022)
	Unnamed	*Ephestia kuehniella*	Lewis et al. (2011)
B	wBol1	*Hypolimnas bolina*	Hornett et al. (2010)
	wPipMol	*Culex molestus*	Pinto et al. (2013)
	wPip	*Culex quinquefasciatus*	Klasson et al. (2008)
	wCcep_B_BJ	*Bemisia tabaci*	Hu and Li (2015)
	w1	*Tetranychus urticae*	Suh et al. (2015)
	w2	*Tetranychus urticae*	Suh et al. (2015)
	wAlbB	*Aedes albopictus*	Beebe et al. (2021)
Unknown	ST41	*Zizeeria maha*	Sumi et al. (2017)
	Unnamed	*Laodelphax striatellus*	Yoshida et al. (2019)
	Unnamed	*Tetranychus urticae*	Breeuwer (1997)
		*Tetranychus turkestani*	
	Unnamed	*Haplodiploid thrips*	Nguyen et al. (2017)
	Unnamed	*Glossina morsitans*	Alam et al. (2011)
	Unnamed	*Cotesia sesamiae*	Mochiah et al. (2002)
	Unnamed	*Laodelphax striatellus*	Noda et al. (2001)
		*Sogatella furcifera*	
	wCc	*Terrestrial crustacean*	Moret et al. (2010)
	Unnamed	*Plodia interpunctella*	Sasaki (2009)
		*Ephestia cautella*	
		*Ephestia kuehniella*	

To study the history of *cif* gene origination, a total of 58 strains were used to calculate the synonymous mutation rates (*K_s_*) of the *cifA* and *cifB* genes within each pair of *Wolbachia* strains (Figure 4B). Interestingly, two independent insertion events may have occurred in *Wolbachia* supergroup A, in which an ancient insertion event involved an independent clade (including wNfe, wNfla, wNleu and wNpa strains) and a recent insertion event involved another clade containing almost all supergroup A strains. The phylogenetic analysis of the *cifB* gene showed that there were two different clades in supergroup A, in which the *cifB* genes of four strains (wNfe, wNfla, wNleu, and wNpa) were more ancient (Figure 4C). Further analysis revealed that the physical distance between the *cifA* and *cifB* genes was 53 bp during the ancient insertion event, and the coding sequence (CDS) lengths of the *cifB* genes in the four strains were completely consistent (Supplementary Table S3). In contrast, the physical distance between the two *cif* genes was 75 bp during the recent insertion event, and the CDS length of the *cifB* genes was twice that in the ancient insertion event (Supplementary Table S3). The sequence similarity analysis of the intergenic region between the *cifA* and *cifB* genes showed that the intergenic region sequences involved in each insertion event shared high identity, suggesting that there were distinct haplotypes in the two insertion events (Figure 4D). The *cifB* gene sequences showed a distinct motif composition between the two insertion events (Figure 4E; Supplementary Figure S6). Further analysis showed that the two insertion segments were located in different genome regions in the two lineages of *Wolbachia* strains based on the analysis of microsynteny (Supplementary Figure S7). However, the fragments that contained both *cifA* and *cifB* were inserted at the same location in the *Nomada*-associated *Wolbachia* strains (Figure 4F), and the same pattern was found in the *Wolbachia* strains with the recent insertion (Figure 4G). The results showed that *Wolbachia* strains with the ancestral insertion were only identified in host insects of the genus *Nomada* within Hymenoptera, while the recent insertion was detected in insects belonging to Diptera, Lepidoptera, and Coleoptera (Supplementary Table S3).

Furthermore, more than 52.9% (18 of 34) of supergroup B strains may have lost the *cifA* and *cifB* genes, whereas the corresponding proportion among supergroup A strains was only 13.7% (7 of 51). In contrast to supergroup B strains, the retained *cif* genes of supergroup A strains were highly conserved and displayed lower mean nucleotide diversity (*π* = 0.10371 and 0.08647 in *cifA* and *cifB*, respectively). However, the *cif* genes of supergroup B strains showed a markedly higher evolutionary rate (*π* = 0.11429 and 0.1455 in *cifA* and *cifB*, respectively) than those in supergroup A strains. It is noteworthy that despite the conservation of *cif* gene order, the functional domains of these genes in supergroup B strains showed extensive divergence and differences, in which most important domains were lost (Supplementary Figure S8). In addition, both the synonymous (*K_s_*) and nonsynonymous (*K_a_*) mutation rates of *cif* genes in supergroup B strains were significantly higher than those in supergroup A strains (Figure 4H). This result suggested that during the independent evolution of supergroup A and B strains, the rapid evolution of *cif* genes in supergroup B strains resulted in the loss of their function, reflected in a decrease in the proportion of induced CI strains.

## Discussion

### The small-scale gene duplications in supergroup A and B strains

Here, we present a phylogenetic hypothesis for *Wolbachia* supergroups A, B, C and D based on the analysis of whole genome single copy gene and five housekeeping genes. Our findings indicate that the *Wolbachia* genomes have a complex evolutionary history, including ancient duplication events (Figure 1E) in the ancestor of the four supergroup (A, B, C and D) and a recent duplication event that were occurred in the ancestor of supergroup A and B. These recent duplication events generated abundant overretentive genes related to functions including the synthesis/metabolism of important compounds and the response to stimuli and chemicals, which are important for the diversity of gene functions and adaptation to changing environments. In addition, we found that the genes related to growth and development (Pratt, 1998; Sánchez-Romero et al., 2015) were significant expansion both in supergroup A and B, such as ATPase family associated with various cellular activities, DNA methylase, heat shock protein 90, DNA mismatch repair protein MLH1, cysteine protease and so on, which were perhaps significantly increase the gene repertoires and the genome complexity and could provide a greater chance for natural selection to generate a novel function (Long et al., 2003; Zhang, 2003; Conant and Wolfe, 2008; Lynch et al., 2008; Lipinski et al., 2011; Gao et al., 2017). So, we speculate that extensive gene fractionation occurred during the evolutionary history of arthropod-infecting *Wolbachia* strain genomes, which promoted the retention of genes that are essential for survival and host adaptation. In contrast, nematode-infecting *Wolbachia* strains have established long-lasting mutualistic relationships with their specific hosts (Darby et al., 2012; Godel et al., 2012), leading to a stable state of the genome that may lead to the loss of large numbers of genes (Gerth et al., 2014) or to form species-specific novel genes (Weyandt et al., 2022), as recently reported in another *Wolbachia* study.

### Adaptive evolution of supergroup B strains to a broad host range

Even under perfect transmission fidelity, *Wolbachia* would have limited chances of spreading. In addition, deleterious fitness effects and imperfect transmission impose further restrictions on the spread of *Wolbachia* within a population (Sanaei et al., 2021). Consequently, without the induction of a phenotype driving its the spread of *Wolbachia*, the bacteria may easily be lost from a new host species (Sanaei et al., 2021). In this study, we identified multiple gene duplication events (Figure 1E) in the ancestor of *Wolbachia* A and B strains, which resulted in many gene redundancies in those genomes. Following duplication, the effect of purifying selection on any one duplicated gene is relaxed (Cheng et al., 2018), permitting the loss or differentiation of duplicated genes and regulatory elements (Conant and Wolfe, 2008). Further analysis revealed clear differences in nucleotide diversity, genomic structural mutations, rapidly evolving gene families and functional gene diversity within each *Wolbachia* B strain. This high rate variability may be not due to *Wolbachia* but rather due to peculiar genetic selection in its hosts. From an evolutionary viewpoint, these genetic variations can all be explained as adaptations enhancing bacterial fitness through the fitness of the infected host, which is straightforward in the case of direct positive effects, such as protection against pathogens or nutrient provision (Sanaei et al., 2021). Overall, the random genetic drift of *Wolbachia* strains may promote their adaptability to widespread hosts and may provide direct fitness benefits to their hosts. Therefore, we hypothesize that supergroup B strains responded to host selection *via* rapid genomic and genic evolution, a high degree of instability, and recurrent rearrangements and recombination events (Lo et al., 2007) to adapt to new hosts and achieve large-scale spreading after the divergence of supergroups A and B.

### Origin and evolution of *Wolbachia* cif genes

Based on comparative and transgenic approaches, two differentially genes (*cifA* nad *cifB*) of prophage WO from *Wolbachia* strain wMel recapitulate and enhance cytoplasmic incompatibility (Lindsey et al., 2018). In this study, based on the comparative genomic strategy between *Wolbachia* supergroups A and B, we found that two distinct haplotypes of supergroup A strains were detectable based on the analysis of *cifA*, *cifB* and intergenic sequences, suggesting that there may have been two independent horizontal gene transfer events involving prophage WO. The lineage consisted of wNfe, wNfla, wNleu and wNpa strains with the same haplotype and same insertion position, in which the inserted fragment from the prophage genome may have appeared in the common ancestor of this lineage. This also suggested that the *cif* genes were not present in the last common ancestor of supergroup A strains but rather that they were acquired independently by *Nomada*-associated *Wolbachia*. In contrast, the *Wolbachia* strains of a more recently diverged lineage presented another identical haplotype, and they showed an almost identical insertion location, suggesting that the insertion event may have occurred in the ancestor of the lineage, possibly before the divergence of each strain. Regrettably, complete sequences were not available for the *Wolbachia* strains in other clades, which made it inconvenient to identify the location of the insertion fragment of prophage WO. We believe that the publication of more complete sequences of *Wolbachia* strains will be helpful to systematically study the origin and evolution of *cif* genes in different supergroups.

## Conclusion

In this study, we aimed to reconstruct the evolutionary history, address host adaptation-related evolution and explore the origin and divergence of CI genes in each *Wolbachia* supergroup. Our results thus not only provide a basis for further exploring the evolutionary history of *Wolbachia* adaptation under host selection but also reveal a new research direction for studying the molecular regulation of *Wolbachia*-induced cytoplasmic incompatibility.

## Data availability statement

The original contributions presented in the study are included in the article/Supplementary material, further inquiries can be directed to the corresponding author.

## Author contributions

Y-SR, BL, and C-YS were responsible for comparative genome analysis. BL and Y-SR draft the paper. D-HZ, BL, and YA coordinated the project. All authors contributed to the article and approved the submitted version.

## Funding

The work was funded by the National Key Research and Development Program of China (Grant No. 2018YFE0127100), Shenzhen Science and Technology Program (Grant No. KQTD20180411143628272 and JCYJ20190813144407666) and science technology innovation and industrial development of Shenzhen Dapeng New District (Grant No. KJYF202001-03).

## Conflict of interest

The authors declare that the research was conducted in the absence of any commercial or financial relationships that could be construed as a potential conflict of interest.

## Publisher’s note

All claims expressed in this article are solely those of the authors and do not necessarily represent those of their affiliated organizations, or those of the publisher, the editors and the reviewers. Any product that may be evaluated in this article, or claim that may be made by its manufacturer, is not guaranteed or endorsed by the publisher.

## Footnotes

^1^ https://www.ncbi.nlm.nih.gov/

^2^https://www.omicshare.com/

^3^ https://pubmlst.org/

^4^ https://www.ncbi.nlm.nih.gov/

^5^ http://meme-suite.org/
