# Supplementary material for: Pangenomic analysis of Wolbachia provides insight into the evolution of host adaptation and cytoplasmic incompatibility factor genes
Source: Front Microbiol. 2023 Feb 3;14:1084839. doi: 10.3389/fmicb.2023.1084839 (PMC9937081; doi:10.3389/fmicb.2023.1084839)
Supplement: Supplementary file 4 [file Data_Sheet_1.PDF]

Pangenomic analysis of *Wolbachia* provides insight into the evolution of host adaptation and cytoplasmic incompatibility factor genes

Bo Liu[1, 2*], Ye-Song Ren[1*], Cheng-Yuan Su[1], Yoshihisa Abe[3], Dao-Hong Zhu[1†]

[1] Laboratory of Insect Behavior & Evolutionary Ecology, College of Life Sciences, Central South University of Forestry and Technology, Changsha 410004, China

[2] Shenzhen Branch, Guangdong Laboratory of Lingnan Modern Agriculture, Genome Analysis Laboratory of the Ministry of Agriculture and Rural Affairs, Agricultural Genomics Institute at Shenzhen, Chinese Academy of Agricultural Sciences, Shenzhen, China

[3] Faculty of Social and Cultural Studies, Kyushu University, 744 Motooka, Fukuoka 819-0395, Japan

[*] These authors contributed equally to this work.

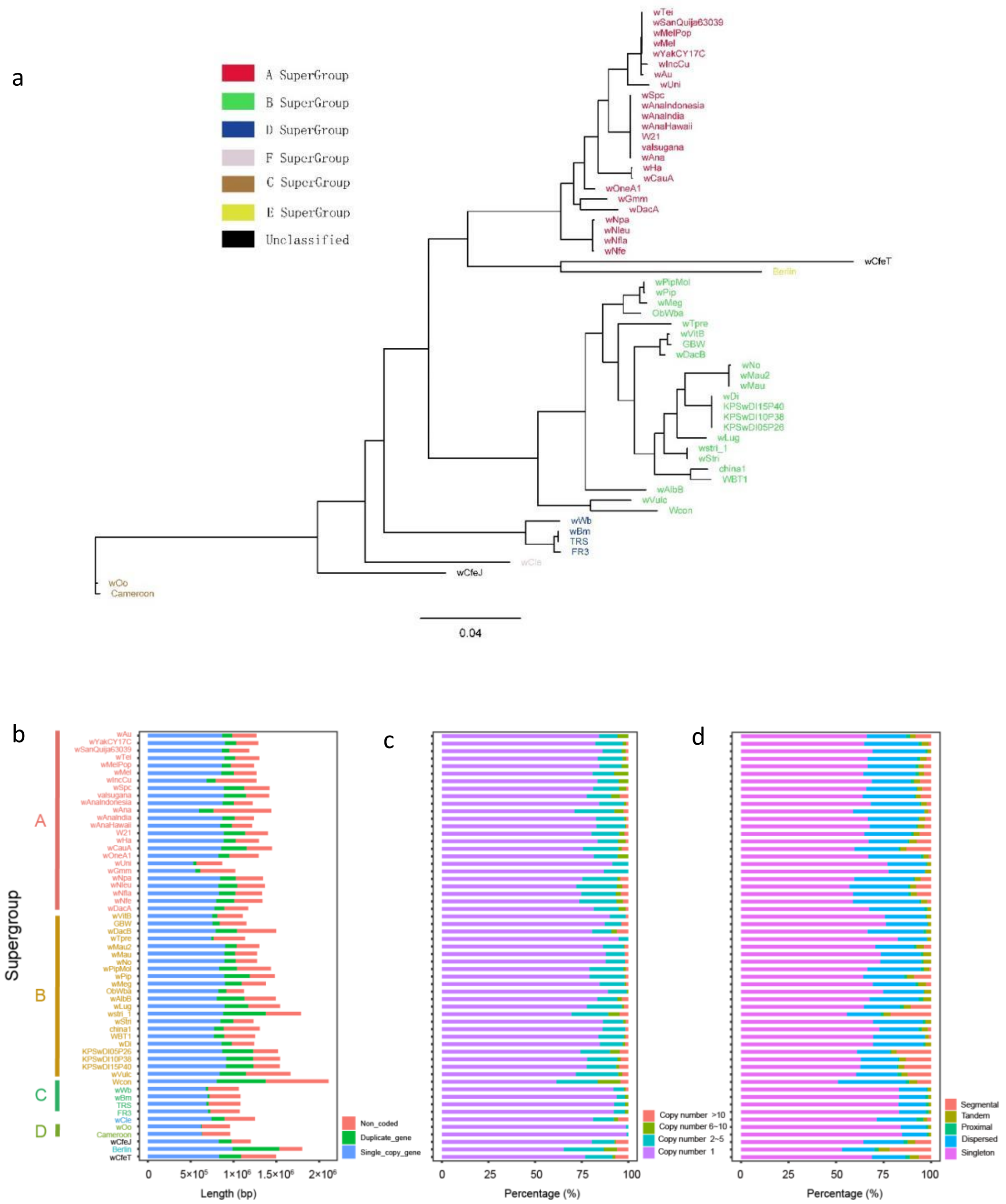

Supplementary Figure S1 (a) The maximum likelihood (ML) tree of *Wolbachia* strains in different supergroups by the nucleotide sequences of five housekeeping genes (gatB, coxA, hcpA, ftsZ, fbpA). Node support was estimated with 100 bootstrap replicates. (b) Total length of single copy gene, duplicated gene and non-coding sequences in each *Wolbachia* strains. (c) The proportion of different copies of the gene in each *Wolbachia* strains. (d) The proportion of genes in different duplicated types in each *Wolbachia* strains.

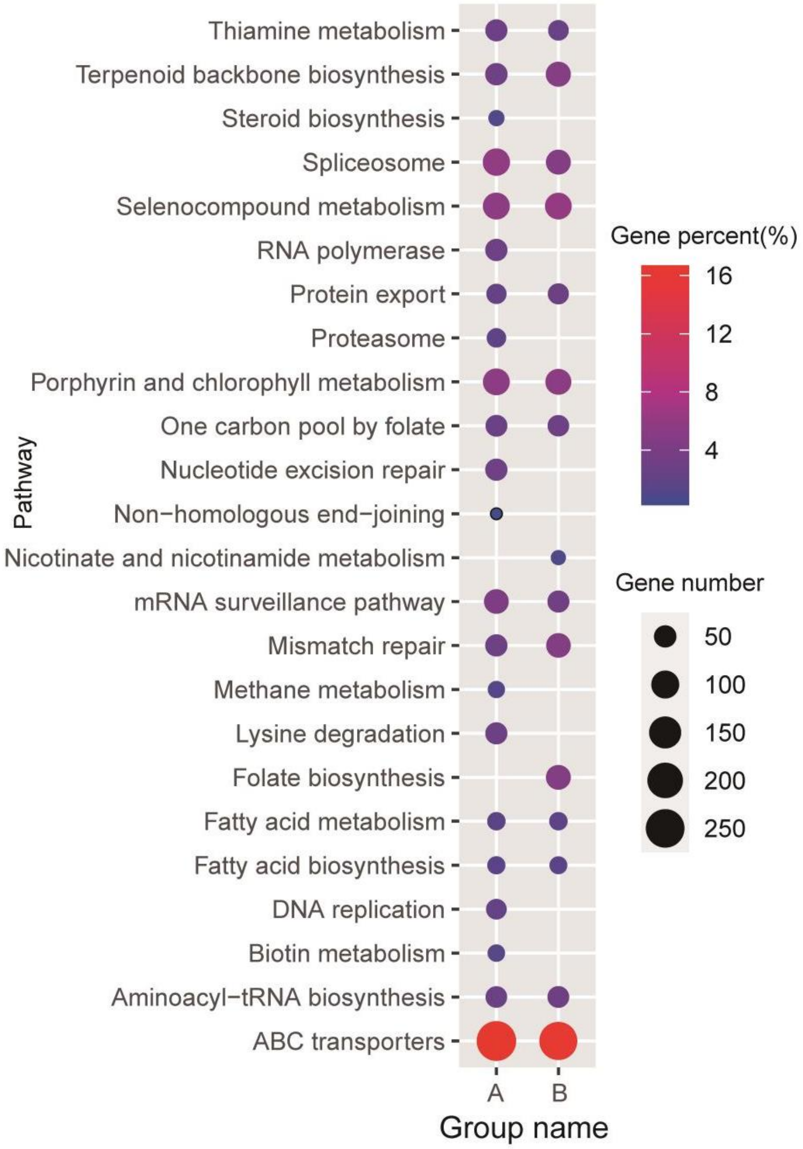

Supplementary Figure S2 The KEGG enrichment of duplicated genes in *Wolbachia* supergroups A and B, respectively.

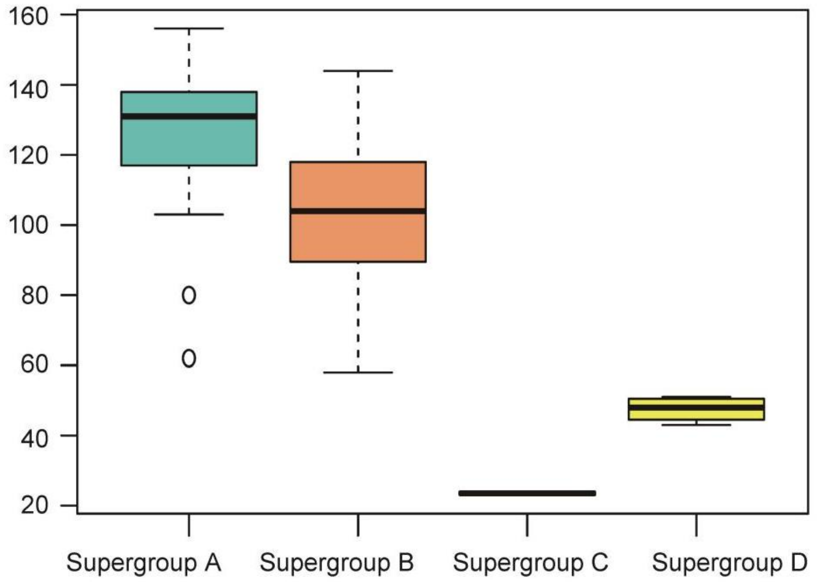

Supplementary Figure S3 Comparison of the number of expanded orthologous groups between arthropod-infecting *Wolbachia* groups (supergroups A and B) and nematode-infecting groups (supergroups C and D).

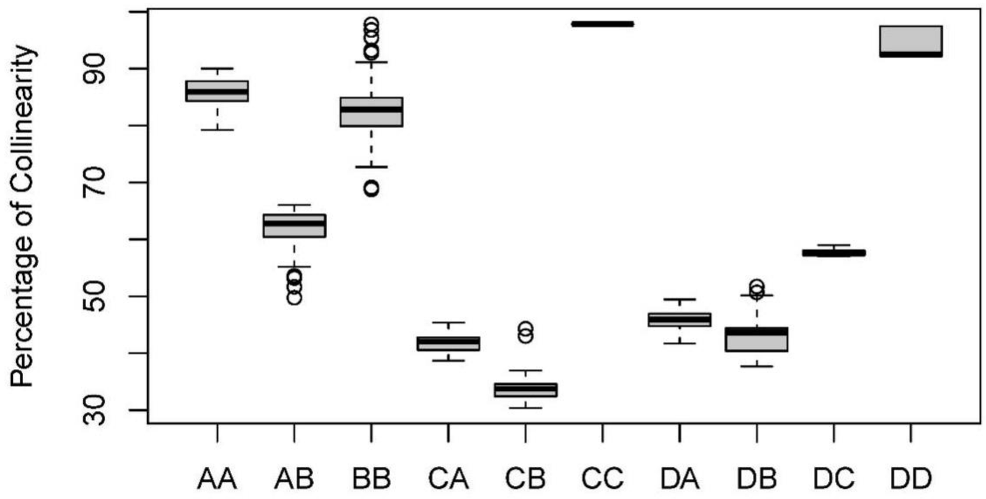

Supplementary Figure S4 The whole genome collinearity analysis of inter- and intra- *Wolbachia* supergroups. A, B, C and D indicate the different *Wolbachia* supergroups.

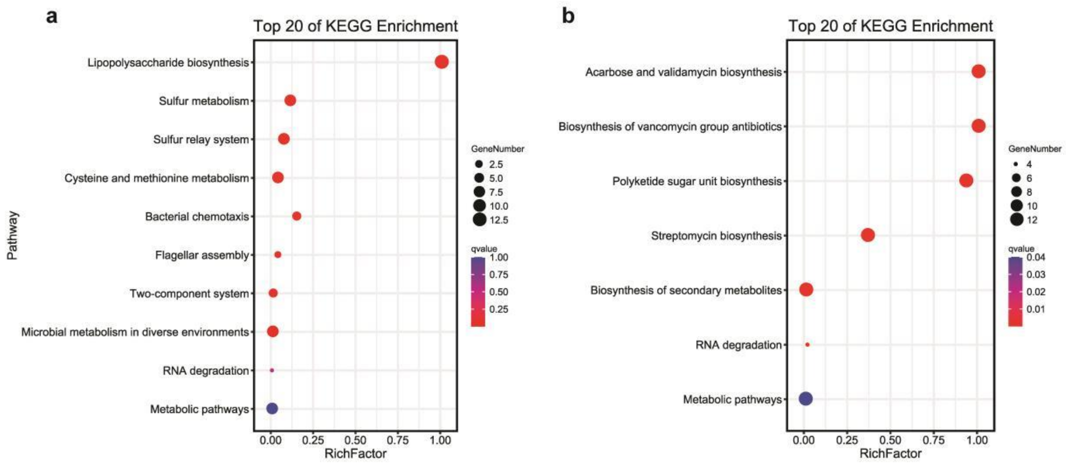

Supplementary Figure S5 The KEGG enrichment of rapidly evolving gene in *Wolbachia* supergroups A **(a)** and B **(b)**, respectively.

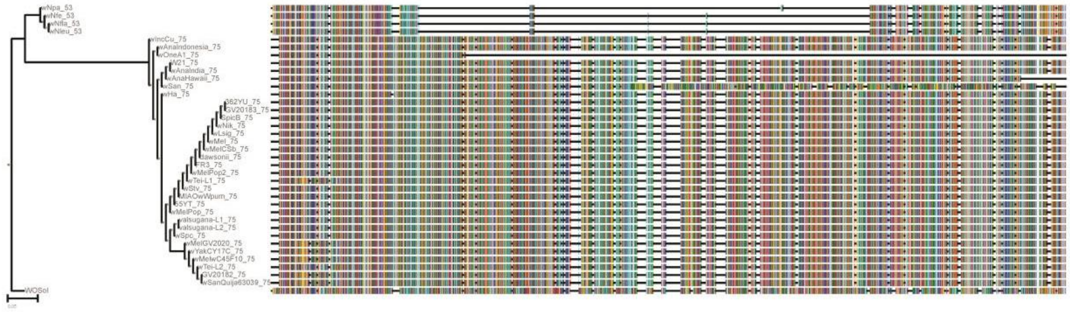

Supplementary Figure S6 The phylogenetic tree and nucleotide multiple sequence alignments of *cifB* in *Wolbachia* strains with different phage insertion (ancient and recent insertion).

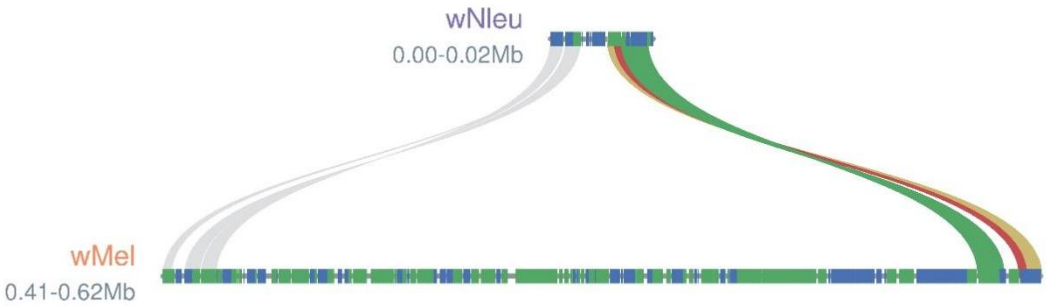

Supplementary Figure S7 Micro-syntenic comparison of representative *Wolbachia* strains with ancient (wNleu strain) and recent (wMel strain) insertion fragments.

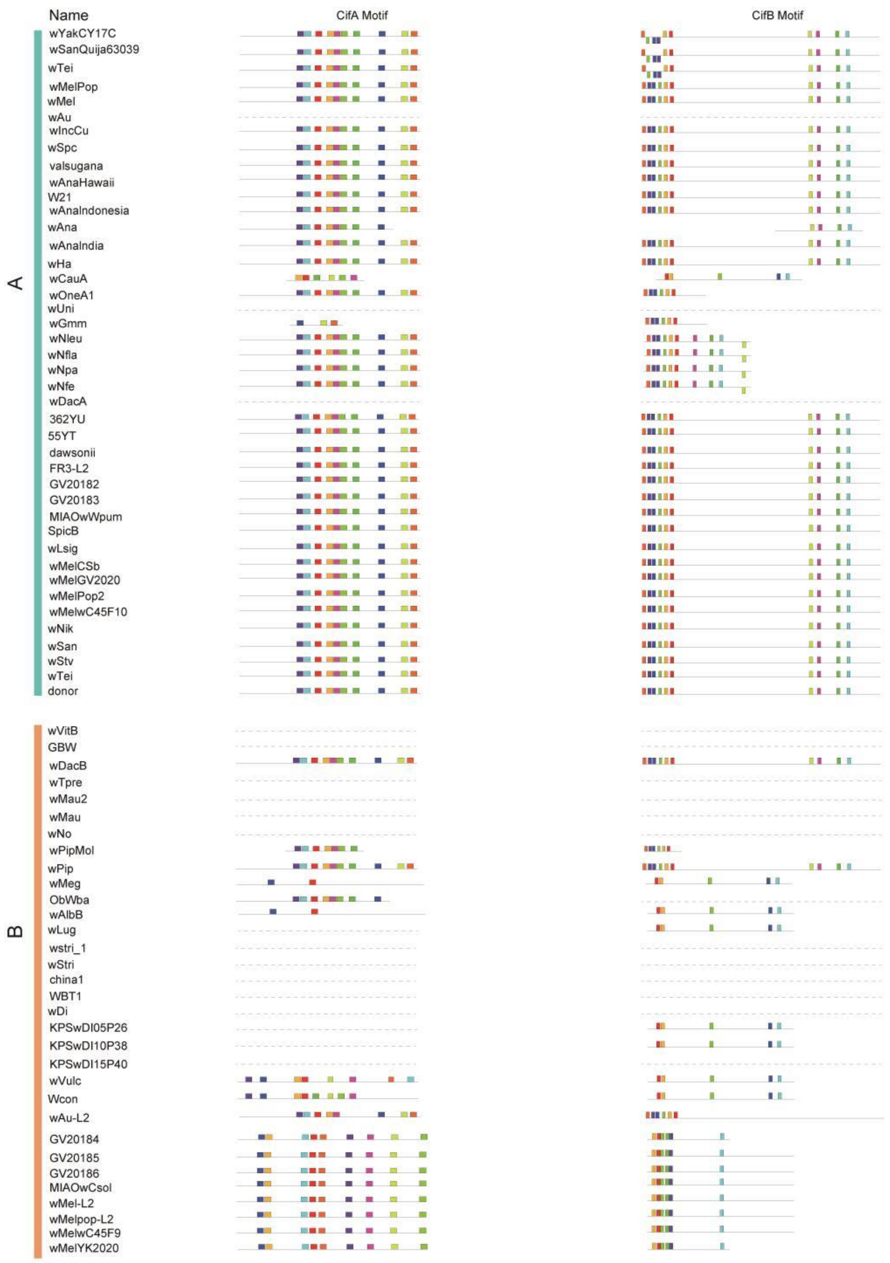

Supplementary Figure S8 The motif composition of *cif* genes in *Wolbachia* supergroups A and B strains, respectively. A and B indicate the *Wolbachia* supergroups A and B. Different colored boxes represent different motif types. The dash lines indicate that the missing of *cif* gene.
